# 3D-QSAR-Based Pharmacophore Modeling, Virtual Screening, and Molecular Docking Studies for Identification of Tubulin Inhibitors with Potential Anticancer Activity

**DOI:** 10.1155/2021/6480804

**Published:** 2021-08-24

**Authors:** Salimeh Mirzaei, Razieh Ghodsi, Farzin Hadizadeh, Amirhossein Sahebkar

**Affiliations:** ^1^Department of Medicinal Chemistry, Faculty of Pharmacy, Hormozgan University of Medical Sciences, Bandar Abbas, Iran; ^2^Department of Medicinal Chemistry, School of Pharmacy, Mashhad University of Medical Sciences, Mashhad, Iran; ^3^Biotechnology Research Center, Pharmaceutical Technology Institute, Mashhad University of Medical Sciences, Mashhad, Iran; ^4^Applied Biomedical Research Center, Mashhad University of Medical Sciences, Mashhad, Iran; ^5^School of Pharmacy, Mashhad University of Medical Sciences, Mashhad, Iran

## Abstract

In this study, we aimed to develop a pharmacophore-based three-dimensional quantitative structure activity relationship (3D-QSAR) for a set including sixty-two cytotoxic quinolines (1-62) as anticancer agents with tubulin inhibitory activity. A total of 279 pharmacophore hypotheses were generated based on the survival score to build QSAR models. A six-point pharmacophore model (AAARRR.1061) was identified as the best model which consisted of three hydrogen bond acceptors (A) and three aromatic ring (R) features. The model showed a high correlation coefficient (*R*^2^ = 0.865), cross-validation coefficient (*Q*^2^ = 0.718), and *F* value (72.3). The best pharmacophore model was then validated by the Y-Randomization test and ROC-AUC analysis. The generated 3D contour maps were used to reveal the structure activity relationship of the compounds. The IBScreen database was screened against AAARRR.1061, and after calculating ADMET properties, 10 compounds were selected for further docking study. Molecular docking analysis showed that compound STOCK2S-23597 with the highest docking score (-10.948 kcal/mol) had hydrophobic interactions and can form four hydrogen bonds with active site residues.

## 1. Introduction

Cancer is a disease identified by the uncontrolled proliferation of cells. As cancer is the second deadliest disease worldwide, discovering new methods and drugs to treat this disease is very important [1, 2]. Nowadays, scientists are looking to find many novel targets for developing anticancer drugs, due to the critical need for new anticancer agents. Microtubules are polymers composed of *α*- and *β*-tubulin heterodimers [3]. They control several cellular functions including motility regulation, cell signaling, secretion, and cell architecture in interphase [4, 5]. Thereby, tubulin and microtubules are important targets for antitumor therapy [6]. The *α*- and *β*-tubulin heterodimers are in dynamic equilibrium with microtubules. The impairment of the dynamic equilibrium of microtubules leads to mitotic arrest and accordingly apoptosis [7, 8]. Therefore, microtubule targeting agents that interfere with dynamic microtubules can be effective in the treatment of cancer. In general, the binding sites for paclitaxel, vinblastine, and colchicine are well determined in tubulin [9–11].

Agents that bind to the vinca alkaloid binding site (e.g., Vincristine) or the colchicine binding site (e.g., colchicine and podophyllotoxin) are determined as microtubule destabilizing agents or inhibitors of tubulin assembly. In contrast, agents that bind to the paclitaxel binding site (e.g., paclitaxel) are tubulin promotors or microtubule stabilizing agents [12, 13].

Antimitotic agents such as vinca alkaloids and taxanes have been used for the clinical treatment of different cancerous patients over the past decades [14, 15]. However, the use of these agents is limited due to drug resistance and associated side effects [16, 17]. Recently, different small molecules have been designed as tubulin inhibitors and anticancer agents [5, 8, 12, 18, 19].

Despite the design and synthesis of promising compounds, antiproliferative and tubulin inhibitory activities of these compounds could not be confirmed until experimentally evaluated, which are time-consuming and expensive besides ethical limitations in using and sacrificing animals. Therefore, computational methods (*in silico* tools) such as pharmacophore modeling, drug screening, and design are essential for activity prediction and greatly reduce the time and cost of drug development [20, 21].

One advantage of *in silico* methodologies is that they can be used to identify new compounds with desirable properties as “druggable” targets before they are synthesized and thus reduce the need for time-consuming and expensive animal and in vitro laboratory work [21, 22].

The relationships between the biological activity and physicochemical properties of a set of compounds could be analyzed with three-dimensional quantitative structure activity relationships (3D-QSARs). QSAR techniques by generating three-dimensional alignment of molecules facilitate design and synthesis of new compounds with superior activity [23]. In this paper, quinolines with cytotoxic activities were used to develop new potent anticancer agents. We selected some cytotoxic quinolines (1-62) as tubulin inhibitors from our previous works [5, 8], generated a training set and test set, and created 279 pharmacophore models with activity prediction ability. Then, we screened the database based on the best pharmacophore model and docked the selected compounds into the colchicine binding site of tubulin. Finally, we selected molecules with the highest docking score as tubulin inhibitor candidates and anticancer agents.

## 2. Materials and Methods

### 2.1. Data Set and Ligand Preparation

For the preparation of common pharmacophore through 3D-QSAR studies, a set including sixty-two quinolines from our previous studies, with cytotoxic activity against A2780 (human ovarian carcinoma) cell line, was selected and the pIC_50_ (pIC_50_ = −logIC_50_) values were calculated [24].

The data set was randomly divided into training and test sets for generation and validation of the model, respectively [25]. The 3D structures of ligands were generated using the builder panel in Maestro and successively optimized using LigPrep module (v4.3, Schrodinger 2016-1) [26]. The energy minimization was done using OPLS_2005 (optimized potentials for liquid simulations) with an implicit distance-dependent dielectric solvation treatment [27].

### 2.2. Pharmacophore 3D-QSAR Modeling

For the generation of pharmacophore and 3D-QSAR models for anticancer agents, Phase (v4.3, Schrodinger 2016-1 was used [28]. The data set ligands were categorized into active, with the threshold of pIC_50_ > 5.5, and inactive, with the threshold of pIC_50_ < 4.7 for the generation of common pharmacophore hypotheses [29, 30].

**Figure 1:**
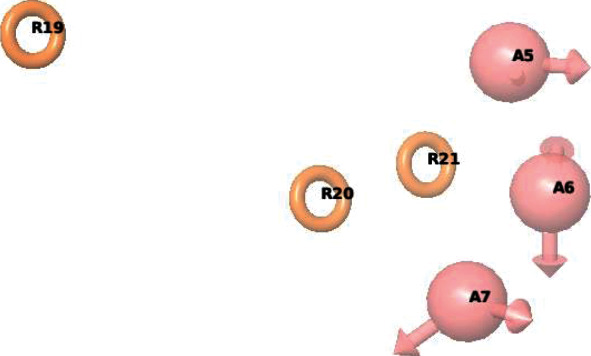
Common pharmacophore hypothesis AAARRR.1061. Pink spheres with vectors A5, AR, and A7 are hydrogen bond acceptor features, and orange open circles R19, R20, and R21 are aromatic ring features.

The default settings were used to generate acceptable conformations, and a maximum of 100 conformers were generated. Alignment was done, and a maximum of one conformer was retained for every ligand. Random selection was used for assigning training and test set for 62 compounds. Twelve (12) compounds were selected as a test set, and the remaining (50 compounds) were used as a training set. Pharmacophore sites were produced based on the pharmacophore features in the Phase module. There are six built-in pharmacological features in Phase, namely, hydrogen bond receptor (A), hydrogen bond donor (D), hydrophobic group (H), negatively charged group (N), positively charged group (P), and aromatic ring (R) [31]. In the generated hypotheses, six common sites were found for all selected compounds. Six of the best resulting hypotheses were scored and ranked by their vector, volume, site scores, survival scores, and survival actives showed in Table 1 [28]. AAARRR.1061 hypothesis which consisted of three hydrogen bond acceptors (A) and three aromatic ring (R) features was selected as the best model for further study (Figure 1).

**Figure 2:**
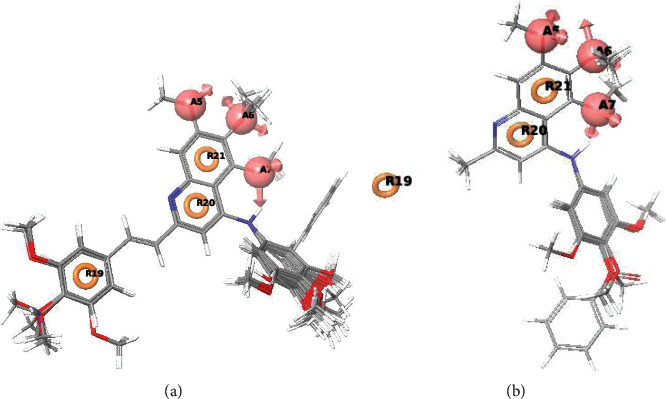
Mapping of the (a) active compounds and (b) inactive compounds on the pharmacophore.

The distance and angles between different sites of the model AAARRR.1061 are shown in Table 2 and Table S1 (supplementary data), respectively. The alignment of active and inactive compounds with the generated common pharmacophore was shown in Figures 2(a) and 2(b). PLS statistical parameters of the model AAARRR.1061 were shown in Table 3. Structure of compounds, experimental and predicted inhibitory activities (pIC_50_ values), residual values, and fitness score of all the ligands were reported in Table 4.

### 2.3. Model Validation

The last step of developing the QSAR model was model validation [32, 33]. The developed pharmacophore hypothesis was validated using potent approaches like the Y-Randomization test and ROC-AUC analysis [34]. The statistical parameters, including the squared correlation coefficient (*R*^2^), cross-validation (leave one out) *Q*^2^, the standard deviation of regression (SD), Pearson's correlation coefficient (Pearson's *R*), statistical significance (*P*), root mean square error (RMSE), and variance ratio (*F*), were shown in Table 3 [35].

#### 2.3.1. Y-Randomization Test

To ensure the validity of our QSAR model, the Y-Randomization technique was performed. The dependent variable vectors were randomly shuffled, and a new QSAR model was developed. The procedure was repeated several times, and the new QSAR models were developed for each random. The resulting *R*^2^ and *Q*^2^ values were compared to that of the original model [36, 37].

#### 2.3.2. ROC-AUC Analysis

To evaluate our hypothesis, receiver operating characteristic (ROC) curve analysis was also performed using the MedCalc statistical software (http://www.medcalc.org). In ROC analysis, the ability of the obtained pharmacophore model was indicated with the area under the curve (AUC) to distinguish a list of compounds as active or inactive compounds in terms of two parameters, sensitivity, and specificity [24].

### 2.4. Drug-Likeness Filtration and Virtual Screening

The IBScreen database containing 211432 compounds was selected. The drug-likeness behavior of the database compounds was predicted by using QikProp version 4.3 (Schrödinger). The compounds were employed for the calculation of pharmacokinetic parameters by QikProp v4.3. Physiochemical descriptors and pharmaceutically relevant properties of compounds were evaluated to analyze druggable properties [27, 38]. Lipinski's rule of five was used to filter the compounds with drug-like properties [39]. To identify the best match molecules, the AAARRR.1061 hypothesis was applied for screening the IBScreen database with drug-like properties. Finally, we selected the compounds with the pIC_50_ value of more than 4 which were considered as the most active compounds, and then, further screening of these compounds is done by ADMET (absorption, distribution, metabolism, elimination, and toxicity) properties using QikProp version 4.3. The compounds complied with Lipinski's rule, with good predicted activity and good ADMET properties, were selected for molecular docking studies [24, 32].

### 2.5. Molecular Docking

**Figure 3:**
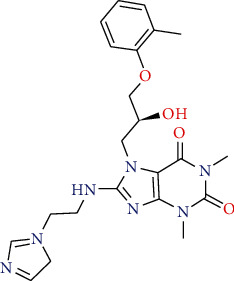
Compound STOCK2S-23597 with the highest docking score.

The docking study was performed using Glide module in Schrodinger suite 2016-1. The crystal structure of tubulin in complex with colchicine (PDB Code: 4O2B) was obtained from protein data bank Brookhaven (*Protein Data Bank (PDB*) at *Brookhaven* National Laboratory). The protein was prepared using the protein preparation wizard in Schrodinger [40]. All the water molecules were deleted, *hydrogen atoms* were added, and energy minimization was performed using the OPLS_2005 force field [26]. The active site was defined with a radius of 15 Å around the ligand present in the crystal structure. The grid box was generated at a centroid of the active site. The compounds were docked into the catalytic domain of tubulin protein (PDB Code: 4O2B) using Grid-based Ligand Docking [41]. The best docked structures were identified using Dock score and Glide energy (Figure S1: supplementary data). The compound STOCK2S-23597 with the lowest docking energy was selected for further studies (Figure 3).

## 3. Results and Discussion

### 3.1. Pharmacophore and 3D-QSAR Models

For the development of the pharmacophore model, the Phase (v4.3) module of Schrodinger 2016-1 was used [28]. All the 62 compounds were randomly divided into a training set (50 compounds), to identify the common pharmacophore hypothesis, and a test set (12 compounds). A maximum of six sites was chosen to find the optimum combination of features to the most active compounds in the data set. In total, 279 common pharmacophore hypotheses were generated using different combinations of variants, such as AAARRR, AAAHRR, AAHRRR, AHHRRR, AHHHRR, and AAHHRR with survival scores ranging between 3.48 and 3.87. Six of the best hypotheses were shown in Table 1.

The best-fitted model AAARRR.1061 consists of three hydrogen bond acceptors (A) and three aromatic ring (R) features, and regression scores of this (AAARRR.1061) pharmacophore hypothesis were further analyzed by PLS in the Phase module using 6 PLS factors (Table 3).

**Figure 4:**
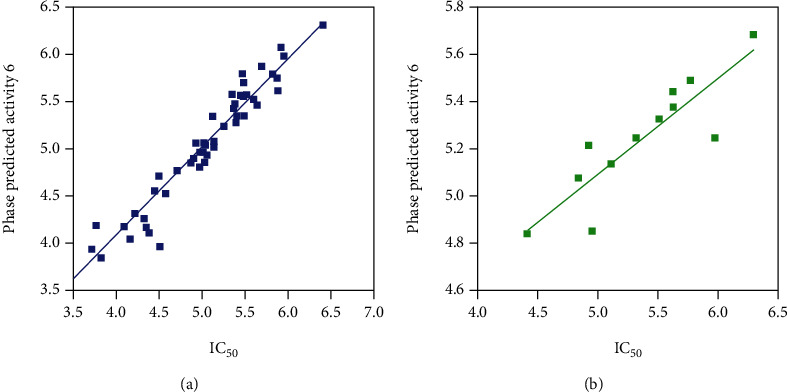
(a) Scatter plot of training set with best-fit line *y* = 0.93*x* + 0.36 (*R*^2^ = 0.93) and (b) test set compounds with best-fit line *y* = 0.41*x* + 3.07 (*R*^2^ = 0.76).

In the regression model, *R*^2^ was used to describe the fitness of data, the correlation coefficient (*Q*^2^) was used to check the external predictability, and the significance of the model was measured by the Fisher ratio (*F*) [31, 36, 37]. Thus, the best QSAR model was chosen based on maximum survival score, good statistical value, good predictive power, and lowest relative conformational energy. Scatter plots for experimental and predicted activities of ligands showed a significant linear correlation of training and test set compounds (Figures 4(a) and 4(b)).

The reliability and predictability of the common pharmacophore model, AAARRR.1061, based on active compounds were determined. The PLS of four was selected as the best model (Table 3). The relevance of the model was displayed by the regression coefficient (*R*^2^ = 0.865) of the training and cross-validation coefficient (*Q*^2^ = 0.718). The stability of the generated models ranged from 0.454 to 0.941. The *F* value was found to be 72.3 and a *P* value of 5.278*e*-019. Moreover, the greater degree of confidence in the model is indicated by Pearson *R* of 0.778. Standard deviation (SD) value of 0.260 and root mean square error (RMSE) of 0.334 showed the ability of the generated model for prediction of unknown compound activity in the test set.

These results demonstrated that not only the pharmacophore model AAARRR.1061 could efficiently estimate the cytotoxic activity of the training set, but it also was fitted for the test set. Hence, AAARRR.1061 was validated to be a reliable pharmacophore mode to recognize cytotoxic agents with the ability to *inhibit* the *tubulin* polymerization, and then, it will be used to screen the database.

### 3.2. Model Validation

#### 3.2.1. Y-Randomization Test

Validation of the model was performed by applying Y-Randomization. We built ten random and repeated all procedures to develop a model (Table S2**)**. All the *R*^2^ and *Q*^2^ of the generated models from random were lower (less than 0.26) compared to the original model. This indicated that our model was not generated by chance.

#### 3.2.2. ROC-AUC Analysis

**Figure 5:**
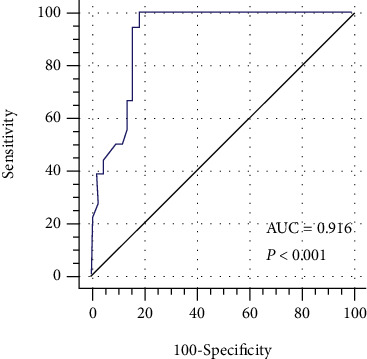
ROC curve obtained by AAARRR.1061 model against random curve.

Additional validation of the common pharmacophore model, AAARRR.1061, was performed using the AUC of the ROC curve. The ROC curve obtained for the validation showed an excellent AUC value of 0.916 (Figure 5), indicating that the model differentiated the active compounds from the inactive ones efficiently (*P* < 0.001). The sensitivity, specificity, and accuracy of the model were 74.67, 86.09, and 80.03%, respectively.

### 3.3. 3D-QSAR Contour Map Analysis

**Figure 6:**
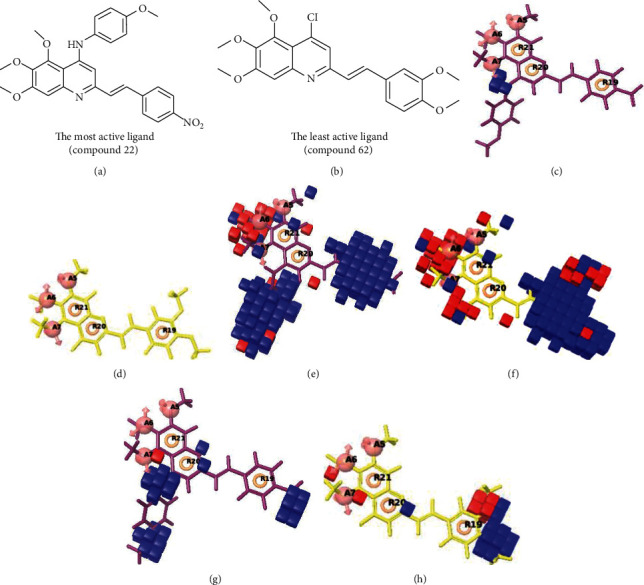
Pictorial representation of the contour maps generated in the context of (c, d) hydrogen bond donor, (e, f) hydrophobic features, and (g, h) electron-withdrawing groups with (a) most active compound 22 and (b) least active compound 62 using the QSAR model.

To analyze 3D-QSAR results, the model was superimposed on the most active ligand (compound 22) and the least active ligand (compound 62). The generated contour plots (Figures 6(a)–6(f)) showed the correlation of structural properties of compounds including electron-withdrawing, hydrophobic, and H-bond donor properties concerning their activity. Blue cubes indicated favorable regions while red cubes indicated unfavorable regions for biological activity [42, 43].

The hydrogen-bond donor nature was compared for the most active compound 22 (Figure 6(a)) and the least active compound 62 (Figure 6(b)). In Figure 6(a), blue cubes were observed at regions lied over the amine group present at position 4 of the quinoline ring. On the other hand, in the least active compound 62 without an amino group at the same steric position (Figure 6(b)), no blue cube was observed in the same region. Therefore, the presence of N-aryl with hydrogen donor amine group was vital for the cytotoxicity and tubulin inhibitory activity. This assumption was further supported by the low activity of compounds 65-71, which do not have N-aryl at position 4 of the quinoline ring.

Figures 6(c) and 6(d) showed the favorable and unfavorable hydrophobic features for the most active compound and least active compound.

Figure 6(c) revealed that the blue cubes were generated around the hydrophobic arylstyryl at position 2 and N-aryl at position 4 of the quinoline core were essential for anticancer activity.

In Figure 6(d), red cubes were generated at position 4 of the quinoline core of the least active compound. In this compound, a chloro substituent was present at this region instead of the hydrophobic N-aryl group. Thus, the results revealed that red colored unfavorable regions at these positions could be responsible for the decrease of activity. This was also confirmed by less activity of compounds 65-71 possessing chloro group at position 4 of the quinoline ring.

In Figure 6(e), blue cubes were observed at the para position of N-aryl indicating the preference of electron-withdrawing groups at this position (the presence of an electronegative atom, such as oxygen or nitrogen, was desirable because of the *inductive electron*-*withdrawing effect*). Also, blue cubes were observed at the para position of the styryl group at position 2 of the quinoline core of the most active compound possessing electron-withdrawing group (NO_2_). It seems that the presence of an electron-withdrawing group at styryl moiety increased the anticancer activity. The high activity of compounds 23-30 and 45-49 possessing NO_2_ and F groups at styryl moiety supports this finding.

### 3.4. Virtual Screening

First, the IBScreen database containing 211432 compounds was selected. Lipinski's rule of five was used to scrutinize these compounds. The applied filter gave a total of 183084 compounds. The validated 3D-QSAR pharmacophore model AAARRR.1061 was used as a 3D structural query for retrieving potent compounds from the IBScreen database [24]. A thousand hits were obtained in this step for a match to hypothesis AAARRR.1061.

Compounds with a pIC_50_ value of more than 4 were selected as the active compounds, and hence, 34 compounds were obtained which were further subjected to filtering by applying ADMET properties. The result showed that 10 hits were retrieved with good ADMET properties. The descriptors used in ADMET prediction and criteria for hit selection were shown in Table S3.

Structures of the top 10 best-fit molecules were shown in figure S1 (supplementary data), and molecular properties and ADMET properties were depicted in Table S3 and S4, respectively.

### 3.5. Molecular Docking

Above 10 hits obtained from the virtual screening process were subjected to molecular docking studies. A tubulin complex with colchicine was chosen as the target protein for molecular docking using the Glide in Schrodinger 2016. Molecular docking studies were performed to predict the binding conformation of the compounds. The top 10 compounds retrieved by ADMET properties were docked into the binding site of tubulin. Binding interactions and binding energies of these 10 molecules with tubulin were shown in Table S5. Dock scores for the screened compounds ranged between -10.948 (STOCK2S-23597) and -5.991 (STOCK2S-05500).

**Figure 7:**
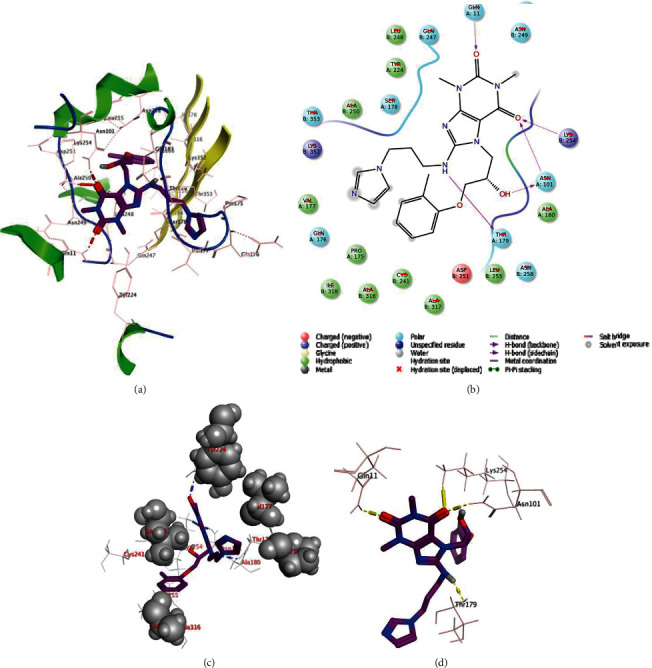
(a) Binding mode, (b) 2D-ligand interaction diagram, (c) hydrophobic interactions, and (d) hydrogen interactions of compound STOCK2S-23597 in the catalytic pocket of 4O2B.

Protein-ligand interactions of compound STOCK2S-23597 with the highest docking score were further analyzed (Figures 7(a)–7(d)). Molecular docking analysis of this compound showed hydrogen bond interactions with four residues of Gln*α* (chain A) 11 (bond length = 2.63 Å), Lys*β* (chain B) 254 (bond length = 2.48 Å), Asn*α*101 (bond length = 2.33 Å), and Thr*α* 179 (bond length = 2.05 Å). Compound STOCK2S-23597 exhibited hydrophobic interactions with the key residues necessary for binding of inhibitors including Leu*β* 248, Tyr*α* 224, Ala*β* 250, Val*α* 177, Ile*β* 318, Cys*β* 241, Ala*β* 316, Ala*β* 317, Ala*α* 180, and Leu*β* 255.

This compound also showed polar interactions with amino acid residues such as Asn*β* 249, Ser*α* 178, Thr*β* 353, Gln*α* 176, and Asn*β* 258.

The binding mode of the most active compound (22) is exhibited in the figure S2. In this compound nitrogen atom of the aniline ring interacted with Thr*α* 179, the methoxy group, interacted with Lys*β* 254, and the nitro group formed a hydrogen bond with Arg*α* 221. These results are consistent with the finding of 3D-QSAR study which showed nitrogen atom of the aniline ring, and the nitro group has key roles in the activity of compound 22.

## 4. Conclusion

In this study, 279 pharmacophore models were generated based on a series of quinolines as anticancer agents and tubulin inhibitors. A six-point pharmacophore model (AAARRR.1061) was identified as the best model which consisted of three hydrogen bond acceptors (A) and three aromatic ring (R) features and was then validated by Y-Randomization test and ROC-AUC analysis. The model showed a high correlation coefficient (*R*^2^ = 0.865), cross-validation coefficient (*Q*^2^ = 0.718), *F* value (72.3), and a *P* value of 5.278*e*-019 at 6 component PLS level. The contour maps obtained for the model with the most active and the least active compounds confirmed that the presence of N-aryl with hydrogen donor amine group at position 4 of the quinoline ring, the arylstyryl hydrophobic group at position 2, and N-aryl at position 4 of the quinoline core and the presence electron-withdrawing group at the para position of arylstyryl group were vital for the cytotoxicity and tubulin inhibitory activity.

AAARRR.1061 was used as a 3D query to screen the IBScreen database, and we obtained 1000 compounds. Compounds with a pIC_50_ value of more than 4 (34 compounds) were selected as the most active compounds. After applying ADMET properties, 10 compounds were selected for further docking studies. Ultimately, compound STOCK2S-23597 with the highest docking score (-10.948 kcal/mol) was selected as a potent tubulin inhibitor. It formed four hydrogen bonds and hydrophobic interactions with tubulin active site residues.

The obtained results suggested that the proposed 3D-QSAR model and hits obtained on virtual screening of the database have provided new chemical starting points for design and development of novel tubulin targeting agents.

## Figures and Tables

**Table 1:** The score of different parameters of the hypotheses.

S. No.	Hypothesis	Survival score	Survival inactive	Site	Vector	Matches	Activity	Inactive
1	AAARRR.1061	3.870	1.270	0.98	0.992	18	5.640	2.599
2	AAAHRR.319	3.863	1.258	0.98	0.991	18	5.895	2.605
3	AAAHRR.311	3.863	1.258	0.98	0.999	18	5.895	2.605
4	AAHRRR.1415	3.862	1.280	0.97	0.999	18	5.640	2.582
5	AAAHRR.327	3.861	1.280	0.97	0.999	18	5.640	2.581
6	AAAHRR.326	3.861	1.280	0.97	0.999	18	5.513	2.851

A: acceptor; H: hydrophobic; R: aromatic ring.

**Table 2:** Intersite distances between the pharmacophoric sites of AAARRR.1061.

Entry	Site1	Site2	Distance (Å)
AAARRR.1061	A5	A6	2.702
AAARRR.1061	A5	A7	4.830
AAARRR.1061	A5	R19	10.134
AAARRR.1061	A5	R20	5.045
AAARRR.1061	A5	R21	2.803
AAARRR.1061	A6	A7	2.832
AAARRR.1061	A6	R19	11.316
AAARRR.1061	A6	R20	5.053
AAARRR.1061	A6	R21	2.778
AAARRR.1061	A7	R19	10.384
AAARRR.1061	A7	R20	3.761
AAARRR.1061	A7	R21	2.808
AAARRR.1061	R19	R20	6.632
AAARRR.1061	R19	R21	8.546
AAARRR.1061	R20	R21	2.423

**Table 3:** PLS statistical parameters of the model AAARRR.1061.

PLS	SD	*R* ^2^	*F*	*P*	Stability	RMSE	*Q* ^2^	Pearson *R*
1	0.4189	0.6288	81.3	6.698*e*-12	0.9411	0.3486	0.5412	0.8756
2	0.3627	0.7276	62.8	5.339*e*-14	0.8681	0.3198	0.6137	0.874
3	0.2995	0.8182	69	4.698*e*-17	0.6923	0.3733	0.4737	0.7448
4∗	0.2606	0.8653	72.3	5.278*e*-19	0.6111	0.334	0.7186	0.7787
5	0.2238	0.9029	81.8	3.836*e*-21	0.5238	0.4009	0.393	0.6463
6	0.1941	0.9286	93.2	5.226*e*-23	0.4545	0.4409	0.2658	0.5743

SD: standard deviation of regression; *R*^2^: regression coefficient; *F*: ratio of the model variance to the observed activity variance (variance ratio); *P*: significance level of variance ratio; *Q*^2^: cross-validated correlation coefficient for the test set; RMSE: the RMS error in the test set predictions. ^∗^Best model.

**Table 4:** Structures and properties of train and test ligands.

Ligand	Structure	Experimental activity (pIC_50_)	Predicted activity (pIC_50_)	Residual activity	Fitness
1	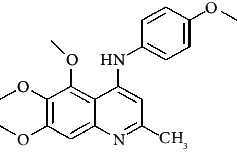	4.415	4.56	-0.145	2.29
2	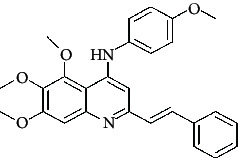	5.122	5.62	-0.498	2.87
3	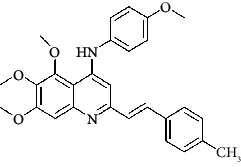	5.339	5.35	0.011	2.90
4	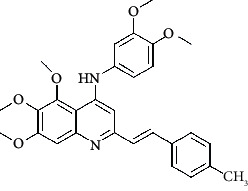	5.513	5.68	-0.167	2.84
5	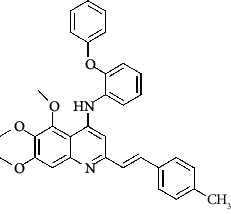	4.961	4.72	0.241	2.71
6^t^	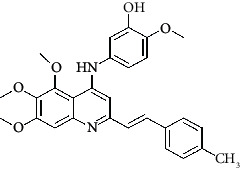	5.633	5.41	0.223	2.86
7^t^	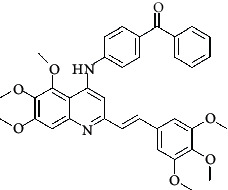	4.845	5.05	-0.205	2.75
8	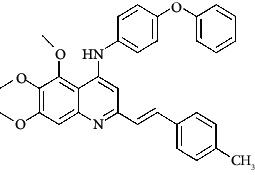	4.922	5.17	-0.248	2.80
9	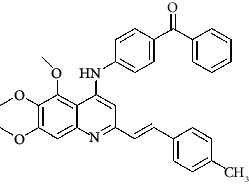	4.526	4.92	-0.394	2.80
10^t^	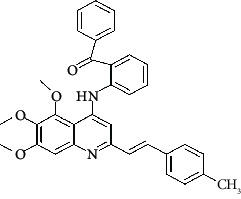	5.140	5.24	-0.1	2.67
11	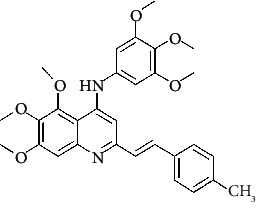	5.879	5.73	0.149	2.83
12	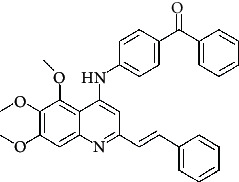	4.178	4.11	0.068	2.76
13^t^	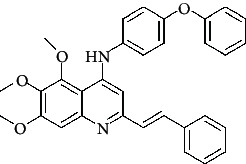	4.970	5.01	-0.04	2.78
14	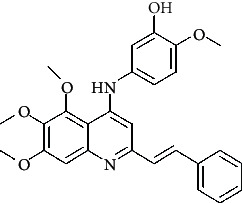	5.660	5.36	0.3	2.88
15	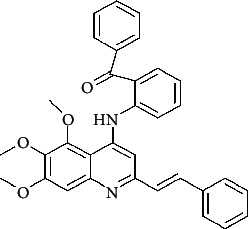	4.992	5.35	-0.358	2.69
16	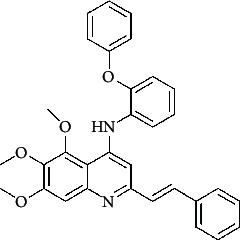	5.037	4.95	0.087	2.68
17	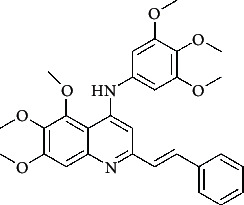	5.479	5.77	-0.291	
18	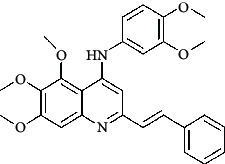	5.254	5.35	-0.096	2.89
19	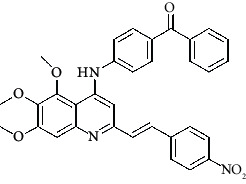	5.423	5.20	0.223	2.80
20	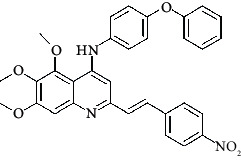	5.038	4.83	0.203	2.78
21	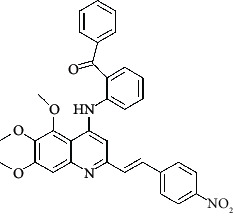	5.015	5.08	-0.065	2.73
22	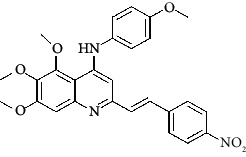	6.414	5.82	0.594	2.89
23	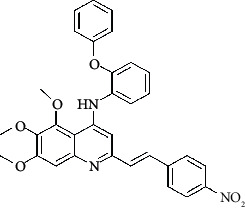	4.734	4.79	-0.056	2.70
24	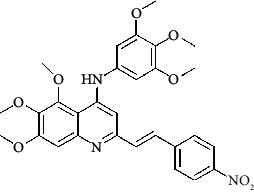	5.498	5.58	-0.082	2.87
25	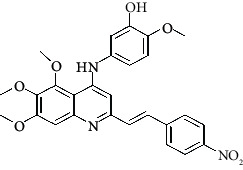	5.129	5.27	-0.141	2.87
26	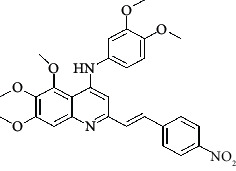	5.840	5.58	0.26	2.91
27^t^	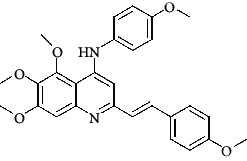	5.356	5.72	-0.364	2.90
28^t^	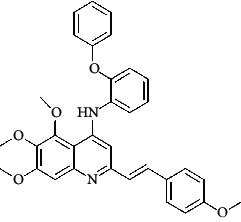	5.056	4.93	0.126	2.65
29^t^	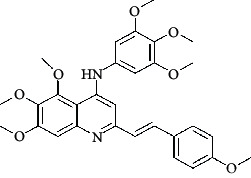	5.376	5.34	0.036	2.88
30	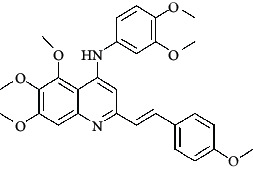	5.460	5.53	-0.07	2.85
31	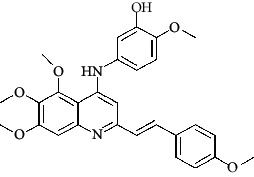	5.612	5.50	0.112	2.87
32	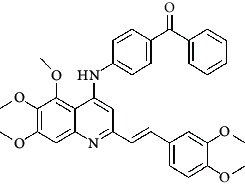	4.885	4.79	0.095	2.82
33^t^	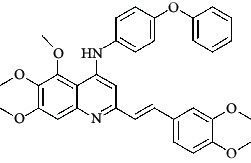	5.393	5.31	0.083	2.81
34	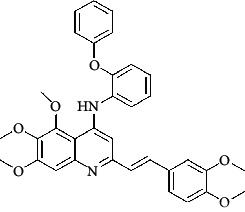	5.149	4.95	0.199	2.73
35^t^	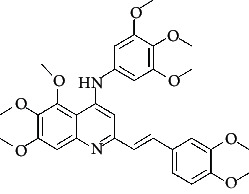	6.297	5.63	0.667	2.90
36	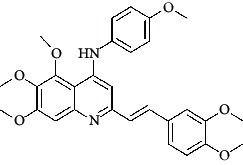	5.986	5.44	0.546	2.96
37^t^	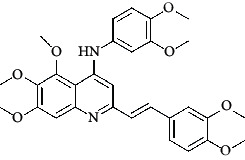	5.768	5.57	0.198	2.91
38^t^	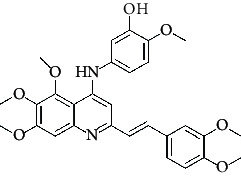	5.640	5.43	0.21	3
39^t^	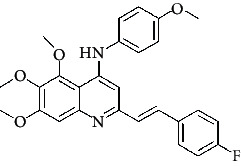	5.492	5.62	-0.128	2.91
40	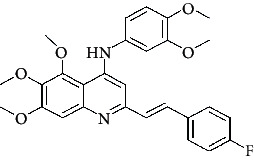	5.895	5.62	0.275	2.91
41	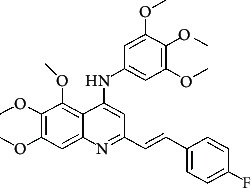	5.963	5.83	0.133	2.83
42	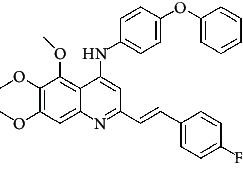	4.930	5.40	-0.47	2.81
43	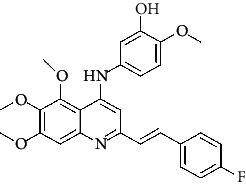	5.931	5.83	0.101	2.87
44	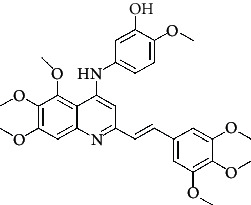	5.708	5.89	-0.182	2.87
45	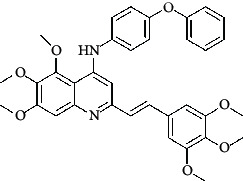	4.594	4.78	-0.186	2.77
46	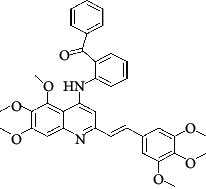	5.504	5.44	0.064	2.67
47	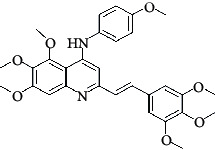	5.403	5.66	-0.257	2.91
48	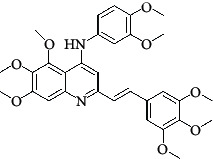	5.539	5.55	-0.011	2.91
49	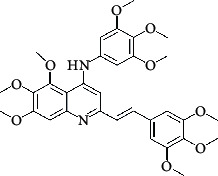	5.824	5.77	0.054	2.89
50	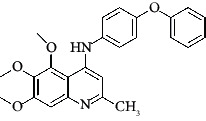	5.074	4.77	0.304	2.21
51	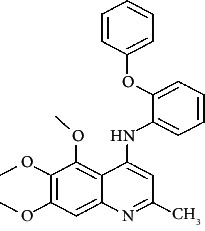	4.919	5.05	-0.131	2.14
52	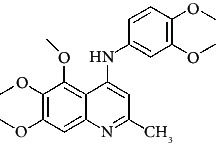	4.234	4.41	-0.176	2.30
53	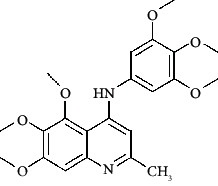	4.095	4.14	-0.045	2.26
54	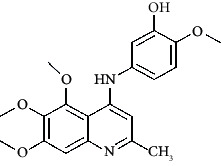	4.459	4.44	0.019	.27
55	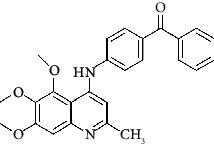	4.342	4.13	0.212	2.19
56	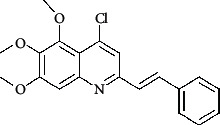	4.516	3.90	0.616	2.67
57	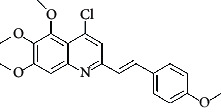	4.399	3.96	0.439	2.69
58	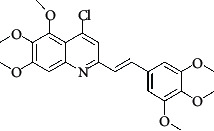	4.319	4.27	0.049	2.71
59	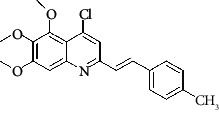	3.830	3.89	-0.06	2.68
60	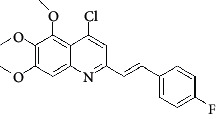	3.734	3.95	-0.216	2.69
61	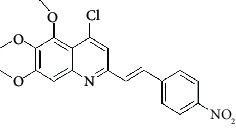	3.771	4.02	-0.249	2.69
62	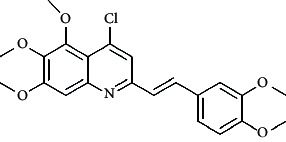	3.127	3.77	-0.643	2.74

^t^Test.

## Data Availability

Data are available upon a reasonable request from the corresponding authors.

## Supplementary Materials

Supplementary Materials Figure S1: structures of top 10 best-fit molecules from the IBScreen database. Figure S2: 2D-ligand interaction diagram of compound 22 in the catalytic pocket of 4O2B. Table S1: intersite angles between the pharmacophoric sites of AAARRR.1061. Table S2: *R*^2^ and *Q*^2^ values after several Y-Randomisation test. Table S3: the molecular property descriptors used in ADMET prediction. Table S4: the descriptors used in ADMET prediction. Table S5: binding interactions of best-fit IBScreen database compounds.
