# Corrigendum to “3D-QSAR-Based Pharmacophore Modeling, Virtual Screening, and Molecular Docking Studies for Identification of Tubulin Inhibitors with Potential Anticancer Activity”

**DOI:** 10.1155/2022/9761279

**Published:** 2022-01-06

**Authors:** Salimeh Mirzaei, Razieh Ghodsi, Farzin Hadizadeh, Amirhossein Sahebkar

**Affiliations:** ^1^Department of Medicinal Chemistry, Faculty of Pharmacy, Hormozgan University of Medical Sciences, Bandar Abbas, Iran; ^2^Department of Medicinal Chemistry, School of Pharmacy, Mashhad University of Medical Sciences, Mashhad, Iran; ^3^Biotechnology Research Center, Pharmaceutical Technology Institute, Mashhad University of Medical Sciences, Mashhad, Iran; ^4^Applied Biomedical Research Center, Mashhad University of Medical Sciences, Mashhad, Iran; ^5^School of Pharmacy, Mashhad University of Medical Sciences, Mashhad, Iran

In the article titled “3D-QSAR-Based Pharmacophore Modeling, Virtual Screening, and Molecular Docking Studies for Identification of Tubulin Inhibitors with Potential Anticancer Activity” [1], the authors identified the errors under Section 3.3 3D-QSAR Contour Map Analysis. The corrected content is shown below.

3.3. 3D-QSAR Contour Map Analysis

To analyze 3D-QSAR results, the model was superimposed on the most active ligand (compound 22 (Figure 6(a)) and the least active ligand (compound 62 (Figure 6(b)). The generated contour plots (Figures 6(c)–6(h)) showed the correlation of structural properties of compounds including electron-withdrawing, hydrophobic, and H-bond donor properties concerning their activity. Blue cubes indicated favorable regions while red cubes indicated unfavorable regions for biological activity [42, 43].

The hydrogen-bond donor nature was compared for the most active compound 22 (Figure 6(c)) and the least active compound 62 (Figure 6(d)). In Figure 6(c), blue cubes were observed at regions lied over the amine group present at position 4 of the quinoline ring. On the other hand, in the least active compound 62 without an amino group at the same steric position (Figure 6(d)), no blue cube was observed in the same region. Therefore, the presence of N-aryl with the hydrogen donor amine group was vital for the cytotoxicity and tubulin inhibitory activity. This assumption was further supported by the low activity of compounds 65-71, which do not have N-aryl at position 4 of the quinoline ring.

Figures 6(e) and 6(f) show the favorable and unfavorable hydrophobic features for the most active compound and least active compound. Figure 6(e) reveals that the blue cubes were generated around the hydrophobic arylstyryl at position 2 and N-aryl at position 4 of the quinoline core were essential for anticancer activity. In Figure 6(f), red cubes were generated at position 4 of the quinoline core of the least active compound. In this compound, a chloro substituent was present at this region instead of the hydrophobic N-aryl group. Thus, the results revealed that red-colored unfavorable regions at these positions could be responsible for the decrease of activity. This was also confirmed by less activity of compounds 65-71 possessing the chloro group at position 4 of the quinoline ring.

In Figure 6(g), blue cubes were observed at the para position of N-aryl indicating the preference of electron-withdrawing groups at this position (the presence of an electronegative atom, such as oxygen or nitrogen, was desirable because of the inductive electron-withdrawing effect). Also, blue cubes were observed at the para position of the styryl group at position 2 of the quinoline core of the most active compound possessing the electron-withdrawing group (NO_2_). It seems that the presence of an electron-withdrawing group at styryl moiety increased the anticancer activity. The high activity of compounds 23-30 and 45-49 possessing NO_2_ and F groups at styryl moiety supports this finding.
