# Supplementary material for: 3D-QSAR-Based Pharmacophore Modeling, Virtual Screening, and Molecular Docking Studies for Identification of Tubulin Inhibitors with Potential Anticancer Activity
Source: Biomed Res Int. 2021 Aug 24;2021:6480804. doi: 10.1155/2021/6480804 (PMC8410400; doi:10.1155/2021/6480804)
Supplement: Supplementary Materials — Figure S1: structures of top 10 best-fit molecules from the IBScreen database. Figure S2: 2D-ligand interaction diagram of compound 22 in the catalytic pocket of 4O2B. Table S1: intersite angles between the pharmacophoric sites of AAARRR.1061. Table S2: R2 and Q2 values after several Y-Randomisation test. Table S3: the molecular property descriptors used in ADMET prediction. Table S4: the descriptors used in ADMET prediction. Table S5: binding interactions of best-fit IBScreen database compounds. [file 6480804.f1.docx]

**Table S1.** Intersite angles between the pharmacophoric sites of AAARRR.1061

| Entry | Site1 | Site2 | Site3 | Angle |
| --- | --- | --- | --- | --- |
| AAARRR.1061 | A6 | A5 | A7 | 30.0 |
| AAARRR.1061 | A6 | A5 | R19 | 109.2 |
| AAARRR.1061 | A6 | A5 | R20 | 74.6 |
| AAARRR.1061 | A6 | A5 | R21 | 60.6 |
| AAARRR.1061 | A7 | A5 | R19 | 79.3 |
| AAARRR.1061 | A7 | A5 | R20 | 44.7 |
| AAARRR.1061 | A7 | A5 | R21 | 30.6 |
| AAARRR.1061 | R19 | A5 | R20 | 34.6 |
| AAARRR.1061 | R19 | A5 | R21 | 48.7 |
| AAARRR.1061 | R20 | A5 | R21 | 14.1 |
| AAARRR.1061 | A5 | A6 | A7 | 121.5 |
| AAARRR.1061 | A5 | A6 | R19 | 57.7 |
| AAARRR.1061 | A5 | A6 | R20 | 74.3 |
| AAARRR.1061 | A5 | A6 | R21 | 61.5 |
| AAARRR.1061 | A7 | A6 | R19 | 63.9 |
| AAARRR.1061 | A7 | A6 | R20 | 47.3 |
| AAARRR.1061 | A7 | A6 | R21 | 60.1 |
| AAARRR.1061 | R19 | A6 | R20 | 16.6 |
| AAARRR.1061 | R19 | A6 | R21 | 3.8 |
| AAARRR.1061 | R20 | A6 | R21 | 12.8 |
| AAARRR.1061 | A5 | A7 | A6 | 28.5 |
| AAARRR.1061 | A5 | A7 | R19 | 73.5 |
| AAARRR.1061 | A5 | A7 | R20 | 70.7 |
| AAARRR.1061 | A5 | A7 | R21 | 30.6 |
| AAARRR.1061 | A6 | A7 | R19 | 102.0 |
| AAARRR.1061 | A6 | A7 | R20 | 99.1 |
| AAARRR.1061 | A6 | A7 | R21 | 59.0 |
| AAARRR.1061 | R19 | A7 | R20 | 3.1 |
| AAARRR.1061 | R19 | A7 | R21 | 43.0 |
| AAARRR.1061 | R20 | A7 | R21 | 40.1 |
| AAARRR.1061 | A5 | R19 | A6 | 13.0 |
| AAARRR.1061 | A5 | R19 | A7 | 27.2 |
| AAARRR.1061 | A5 | R19 | R20 | 25.6 |
| AAARRR.1061 | A5 | R19 | R21 | 14.3 |
| AAARRR.1061 | A6 | R19 | A7 | 14.2 |
| AAARRR.1061 | A6 | R19 | R20 | 12.6 |
| AAARRR.1061 | A6 | R19 | R21 | 1.2 |
| AAARRR.1061 | A7 | R19 | R20 | 1.8 |
| AAARRR.1061 | A7 | R19 | R21 | 12.9 |
| AAARRR.1061 | R20 | R19 | R21 | 11.3 |
| AAARRR.1061 | A5 | R20 | A6 | 31.0 |
| AAARRR.1061 | A5 | R20 | A7 | 64.6 |
| AAARRR.1061 | A5 | R20 | R19 | 119.8 |
| AAARRR.1061 | A5 | R20 | R21 | 16.3 |
| AAARRR.1061 | A6 | R20 | A7 | 33.6 |
| AAARRR.1061 | A6 | R20 | R19 | 150.8 |
| AAARRR.1061 | A6 | R20 | R21 | 14.7 |
| AAARRR.1061 | A7 | R20 | R19 | 175.1 |
| AAARRR.1061 | A7 | R20 | R21 | 48.3 |
| AAARRR.1061 | R19 | R20 | R21 | 136.1 |
| AAARRR.1061 | A5 | R21 | A6 | 57.9 |
| AAARRR.1061 | A5 | R21 | A7 | 118.8 |
| AAARRR.1061 | A5 | R21 | R19 | 117.1 |
| AAARRR.1061 | A5 | R21 | R20 | 149.6 |
| AAARRR.1061 | A6 | R21 | A7 | 60.9 |
| AAARRR.1061 | A6 | R21 | R19 | 175.0 |
| AAARRR.1061 | A6 | R21 | R20 | 152.5 |
| AAARRR.1061 | A7 | R21 | R19 | 124.1 |
| AAARRR.1061 | A7 | R21 | R20 | 91.6 |
| AAARRR.1061 | R19 | R21 | R20 | 32.5 |

**Figure S1:** Structures of Top 10 Best-fit Molecules from IBScreen Database

**Table S2:** *R*^2^ and *Q*^2^ Values After Several Y-Randomisation Test

| **Iteration** | ***R*^2^** | ***Q*^2^** |
| --- | --- | --- |
| 2 | 0.15 | 0.07 |
| 3 | 0.1 | 0.02 |
| 4 | 0.09 | 0.03 |
| 5 | 0.12 | 0.09 |
| 6 | 0.14 | 0.04 |
| 7 | 0.24 | 0.11 |
| 8 | 0.18 | 0.07 |
| 9 | 0.26 | 0.08 |
| 10 | 0.11 | 0.05 |

**Table S3:** The molecular properties Descriptors used in ADMET prediction

| **Predicted physiochemical properties** | **Used Descriptors** | **Ideal Range in 95% of drugs** |
| --- | --- | --- |
| Predicted molecular weight | MW | 311–650 |
| Predicted dipole moment | DM | 0.000–1000.0 |
| Predicted total molecular solvent accessible surface area | SASA | 300.0–1000.0 |
| Predicted hydrophobic SASA | FOSA | 0–750 |
| Predicted hydrophilic SASA | FISA | 7–330 |
| Predicted carbon Pi SASA | PISA | 0–450 |
| Predicted weakly polar SASA | WPSA | 0–175 |
| Predicted octanol/water partition coefficient | QP log PO/W | −2–6.5 |
| Predicted aqueous solubility | QP Log S | −6.5–0.5 |
| Predicted blood–brain partition coefficient | QPLogBB (Cbrain/Cblood) | −3.0–1.2 |
| Predicted apparent MDCK cell permeability in nm/sec | AffyPMDCK | <25 poor, >500 great |
| Predicted apparent Caco-2 cell permeability in nm/sec | AffyPCaco | <25 poor, >500 great |
| Prediction of binding to human serum albumin. | QPLogKhsa | −1.5–1.5 |
| Predicted IC_50_ *in vitro* | Log HERG | Concern below −5 |
| Predicted skin permeability | QP Log KP | −8.0 to −1.0 |

**Table S4:** The Descriptors used in ADMET prediction

| **Molecule** | **MW** | **Dipole** | **SASA** | **FOSA** | **FISA** | **PISA** | **WPSA** | **Volume** |
| --- | --- | --- | --- | --- | --- | --- | --- | --- |
| STOCK2S-09706 | 435.48 | 7.758 | 717.734 | 178.308 | 153.525 | 385.901 | 0 | 1313.352 |
| STOCK2S-23597 | 467.52 | 6.620 | 824.166 | 353.219 | 162.662 | 308.285 | 0 | 1483.306 |
| STOCK2S-17853 | 476.53 | 8.968 | 754.647 | 346.191 | 125.488 | 346.191 | 0 | 1433.008 |
| STOCK2S-11888 | 465.50 | 6.009 | 743.127 | 267.953 | 147.797 | 327.377 | 0 | 1374.020 |
| STOCK2S-14985 | 469.92 | 6.923 | 743.726 | 172.348 | 166.909 | 332.801 | 71.669 | 1363.278 |
| STOCK1S-53938 | 448.48 | 7.781 | 719.785 | 364.737 | 200.597 | 364.737 | 0 | 1348.584 |
| STOCK1S-10792 | 495.53 | 9.663 | 723.149 | 256.395 | 145.082 | 256.395 | 0 | 1408.880 |
| STOCK1S-  09593 | 464.48 | 7.188 | 680.5 | 229.582 | 97.116 | 353.802 | 0 | 1304.103 |
| STOCK1S-  63549 | 493.17 | 7.085 | 698.004 | 327.127 | 52.047 | 318.83 | 0 | 1337.524 |
| STOCK2S-  05500 | 477.50 | 7.186 | 820.375 | 230.978 | 201.029 | 354.28 | 34.086 | 1452.223 |

**Table S4** (continued)

| **Molecule** | **CNS** | **QPlog**  **Po/w** | **QPlogS** | **QPlog**  **HERG** | **QPP**  **Caco** | **QPlog**  **BB** | **QPP**  **MDCK** | **QPlog**  **Kp** | **QPlog**  **Khsa** | **% Human Oral Absorption** |
| --- | --- | --- | --- | --- | --- | --- | --- | --- | --- | --- |
| STOCK2S-09706 | -2 | 3.332 | -4.741 | -6.478 | 346.752 | -1.607 | 157.458 | -2.029 | 0.157 | 95.273 |
| STOCK2S-23597 | -2 | 3.519 | -5.738 | -6.834 | 284.038 | -1.961 | 126.915 | -2.375 | 0.174 | 83.531 |
| STOCK2S-17853 | -2 | 3.691 | -4.909 | -5.882 | 639.589 | -1.300 | 305.177 | -1.875 | 0.224 | 100 |
| STOCK2S-11888 | -2 | 3.383 | -4.775 | -6.260 | 392.956 | -1.623 | 180.253 | -2.034 | 0.138 | 100 |
| STOCK2S-14985 | -2 | 3.747 | -5.479 | -6.364 | 258.884 | -1.604 | 283.522 | -2.463 | 0.273 | 96.887 |
| STOCK1S-53938 | -2 | 2.768 | -4.208 | -6.182 | 339.807 | -1.570 | 154.052 | -2.121 | -0.069 | 84.163 |
| STOCK1S-10792 | -1 | 15.245 | -4.077 | -5.336 | 416.957 | -1.529 | 192.183 | -2.138 | 0.098 | 81.377 |
| STOCK1S-09593 | 0 | 4.258 | -4.764 | -5.674 | 1188.358 | -0.638 | 596.155 | -1.583 | 0.386 | 66.26 |
| STOCK1S-63549 | 0 | 4.869 | -5.227 | -5.624 | 3179.347 | -0.228 | 1727.101 | -0.876 | 0.55 | 55.300 |
| STOCK2S-05500 | -2 | 3.834 | -7.82 | -7.17 | 122.898 | -2.18 | 78.885 | -3.208 | 0.486 | 83.851 |

| **Compound** | **Dock score** | **No of H-bonds** | **Interacting amino acids** | **H-bond distance** | **glide energy** | **Peredict activity** |
| --- | --- | --- | --- | --- | --- | --- |
| STOCK2S-23597 | -10.948 | 4 | GLNA:11  LYSB:254  ASNA: 101  THRA: 179 | 2.63  2.48  2.33  2.05 | -68.262 | 4.469 |
| STOCK2S-09706 | -9.356 | 1 | VALB: 238 | 2.23 | -60.592 | 4.49 |
| STOCK2S-17853 | -8.972 | 0 | - | - | -56.96 | 4.437 |
| STOCK2S-11888 | -8.921 | 3 | LYSB: 254  ASNA: 101  ASNA: 101 | 2.13  2.69  2.12 | -65.535 | 4.38 |
| STOCK2S-14985 | -8.757 | 2 | ASNA: 101  ASNA: 101 | 2.32  2.02 | -61.886 | 4.363 |
| STOCK1S-53938 | -8.353 | 0 | - | - | -47.781 | 4.447 |
| STOCK1S-10792 | -7.667 | 0 | - | - | -61.988 | 4.294 |
| STOCK1S-09593 | -9.235 | 5 | GLNB: 247  GLNA: 11  ASNB: 249  ASNA: 101  THRA: 179 | 2.65  2.49  2.21  2.53  2.25 | -62.304 | 4.514 |
| STOCK1S-63549 | -8.972 | 3 | SERA: 178  ASNA: 101  ASNA: 101 | 2.37  2.24  2.02 | -68.676 | 4.622 |
| STOCK2S-05500 | -5.991 | 4 | GLNB: 247  GLNA: 11  ASNB: 249  ASNA: 101 | 2.06  2.23  2.72  2.67 | -47.763 | 4.385 |

**Table S5:** Binding Interactions of Best-Fit IBScreen Database Compounds


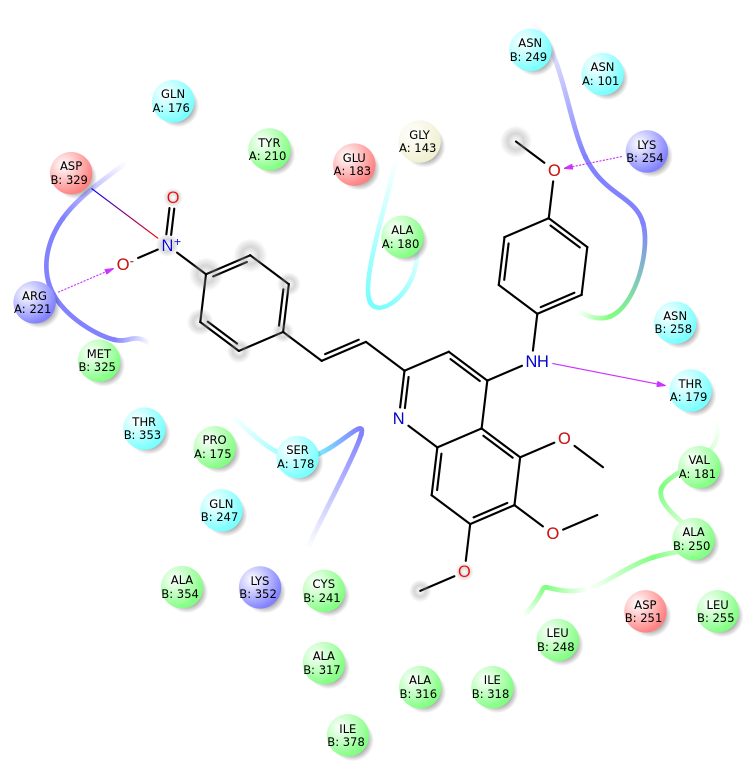


**Figure S2:** 2D-ligand interaction diagram of compound **22** in the catalytic pocket of **4O2B**
